# Delineation of Optimized Single and Multichannel Approximate DA-Based Filter Design Using Influential Single MAC Strategy for Trans-Multiplexer

**DOI:** 10.3390/s24227149

**Published:** 2024-11-07

**Authors:** Britto Pari James, Leung Man-Fai, Mariammal Karuthapandian, Vaithiyanathan Dhandapani

**Affiliations:** 1 Vel Tech Rangarajan Dr. Sagunthala R&D Institute of Science and Technology, Chennai 600062, India; 2 School of Computing and Information Science, Faculty of Science and Engineering, Anglia Ruskin University, Cambridge CB1 1PT, UK; man-fai.leung@aru.ac.uk; 3 Madras Institute of Technology, Anna University, Chennai 600044, India; mariammal@annauniv.edu; 4 National Institute of Technology Delhi, Delhi 110036, India; dvaithiyanathan@nitdelhi.ac.in

**Keywords:** distributed arithmetic, finite impulse response filter, approximate computing, FPGA, multiply-accumulate unit, hardware optimization

## Abstract

In this paper, a multichannel FIR filter design based on the Time Division Multiplex (TDM) approach that incorporates one multiply and add unit, regardless of the variable coefficient length and varying channels, by associating the resource sharing doctrine is suggested. A multiplier based on approximate distributed arithmetic (DA) circuits is employed for effective resource optimization. Although no explicit multiplication was conducted in this realization, the radix-8 and radix-4 Booth algorithms are utilized in the DA framework to curtail and optimize the partial products (PPs). Furthermore, the input stream is truncated with an erratum mending unit to roughly construct the partial products. For an aggregation of PPs, an approximate Wallace tree is taken into consideration to further minimize hardware expenses. Consequently, the suggested design’s latency, utilized area, and power usage are largely reduced. The Xilinx Vertex device is expedited, given the synthesis of the suggested multichannel realization with 16 taps, which is simulated using the Verilog formulary. It is observed that the filter structure with one channel produced the desired results, and the system’s frequency can support up to 429 MHz with a reduced area. Utilizing TSMC 180 nm CMOS technology and the Cadence RC compiler, cell-level performance is also achieved.

## 1. Introduction

Recent technological advancements necessitate dynamic and decisive filter structures, which are predominantly used in communication applications. In earlier studies, filter bank implementation in multiple carrier-modulating designs plays a pivotal role in communication fields [1]. The filter banks perform trans-multiplexing to enhance selectivity and provide required frequency variation with the large dissociation between every subcarrier. Moreover, they can achieve several fast and efficient implementations in addition to major improvements that are well-elucidated in [2], like an improved rejection of narrow-range interferences. Filter banks are also regarded as potential remedies in power line communication (PLC) [3]. An optimization of FIR filters is necessary to execute the multiple carrier modulating design. The multipliers and adders dominate the complexity of filters. By striking a balance between resource utilization and latency, an effective and comprehensive usage of resources throughout time is desired. Owing to the area constraint and logic intricacy, the straightforward realization of P-tap filtering mandates a P multiply and accumulate (MAC) process, and that makes hardware implementation prohibitively expensive. Digital signal processing, or DSP, provides the key to solutions for an extensive range of intricated designs in diverse domains, like multimedia compression, wireless communication, and speech recognition. Large sampling array sizes are now required for many applications, including communication and multimedia, where data from a sole channel may be inaccurate and delayed. As a result, diversified operation of a signal is advised for dependable as well as effective realization. This is due to the advancement of recent DSP technology. To achieve resource optimization, the samples of diversified channel information are efficiently carried out in the filter using a time multiplexing approach [4,5]. Newly with the emergence of Software Defined Radio (SDR), studies have predominantly contemplated the importance of reconfigurability in filters due to the requirement of flexible and curtailed complex design [2,3,4,5,6,7,8,9,10,11,12,13,14,15,16,17,18,19]. Digit-based reconfiguration architecture described in [6] offers a low-power and versatile solution, which is achieved with extensive preciseness and varying filter lengths. Typically, systolic architecture [10], programmable MAC design [6], and programmable shifting method (PSM) [8] are used to build reconfigurable FIR filters. Throughput, power consumption, and hardware complexity are used to evaluate the performances of different designs. Although they need a lot of space, programmable MAC systems use little power and lower supply voltage [6]. When the order of the filter grows, systolic-based architecture increases latency even while it reduces complexity [10]. Because programmable shifters are present, the PSM-based reconfigurable design offers considerable speed improvements and reduced complexity [8]. However, like the rise in filtering lengths, the area enforced increases as well. The use of DA-based approaches [11] has recently grown significantly in popularity, given their high processing throughput and greater speed, which produce computing architectures that are both economical and time- and space-efficient. For DA-oriented calculation, a series of lookup-table (LUT) approaches and shifts with accumulated processes on the LUT output are necessary since impulse response coefficients are assumed to be fixed in the standard DA method, which is possibly used to create the FIR filter with ROM-based LUTs. However, the storage essential for DA-based filter realization grows exponentially with filter order. Systolic disintegration strategies are devised for DA-oriented realization of comprehensive length convolving and larger length filter designs [12] to exterminate the problem of extensive storage requirement. A ROM-based LUT is not suitable for reconfiguration-oriented DA-utilized filters since the filter coefficients vary progressively. With one multiply and accumulate (MAC) unit, the realized filter efficacy with desperate approaches provides good performance [13]. A rewritable RAM-based LUT is required for the design [14]. An alternative method involves employing serial digital-to-analog converters to save the coefficients, resulting in mixed-signal architecture [15]. Additionally, there are numerous publications on DA-based adaptive filter implementations [16,17] in which the coefficients vary on each cycle. Reconfigurability in the MAC channel filter adopts a distinct numbering scheme [20]. Using the DA approach, an effective scheme for the potent sharable-LUT design of reconfiguration utilized filtering [21] is presented, in which the DA units share LUTs for bit slices with varying weights. Furthermore, a very short reconfiguration latency can be attained by progressively modifying the filter accessories during runtime. To lower hardware and power consumption, a lot of academics are focusing on FIR filter architecture. Consequently, an architecture that gets around the stated limitations needs to be identified. In this article, the TDM technique is adopted to realize a multichannel FIR filter with a sole multiply and add modules, irrespective of the number of taps or channels that facilitate using resourcefulness partaking doctrine. For the optimization of resource intricacy of multiply units, the two methodologies, Approximate DA-based radix-4 and radix-8, are implemented. This research proposes an efficient DA design that adapts the radix-8 and radix-4 Booth formulary in an integrated multichannel FIR filter design. This architecture converts the multiply and add operation of weighted sums utilizing multiplier and adder units to a DA design without involving LUTs. Notwithstanding, partial product creation and aggregation blocks are needed even though no multiplier is utilized. The required number of partial products is halved when employing the radix-8 Booth method as opposed to a traditional DA depiction. As a result, the accumulation circuits are significantly reduced. Additionally, an approximation recoded adder is utilized to lower the longest path and utilize area along with power. Also, the truncating procedure is emphasized at the input side to construct the PPs approximately. The proximate Wallace tree is incorporated for the aggregation of PPs to further minimize delay.

The remains of this research are formulated in the forthcoming ways. Decisive approximation-based DA architecture is presented in Section 2. The proposed multichannel FIR schema is highlighted in Section 3. The suggested filter framework efficacy is examined and cited in Section 4. Section 5 consummates the proposed research and further works to be executed.

## 2. Efficient Approximate Distributed Arithmetic FIR (ADA) Filter

Let *X* and *Y* represent the FIR filter’s input and outputs, respectively. A generic form of FIR is expressed as

(1) $$Y\left(n\right)=\sum_{k=0}^{N-1}h_{k}X\left(n-k\right)$$

where *h_k_* is the filter impulse response’s *k*-th coefficient.

**Figure 1:**
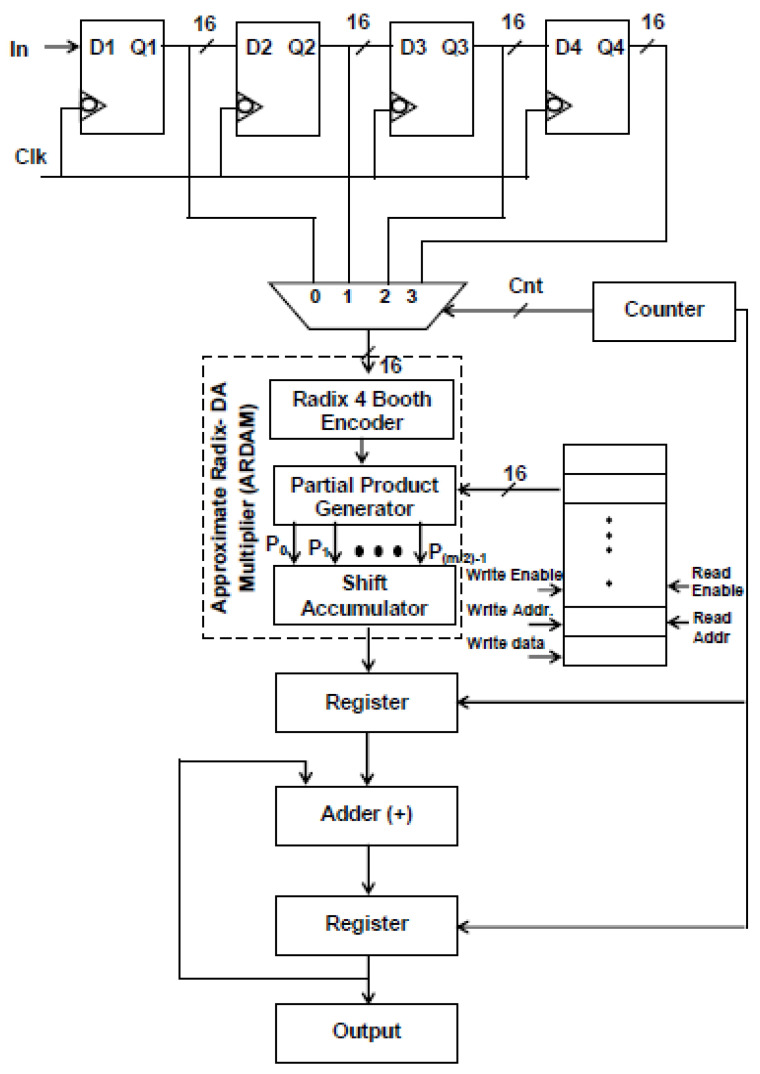
Structure of single ARDAM-based four-tap FIR filter (R-4).

**Figure 2:**
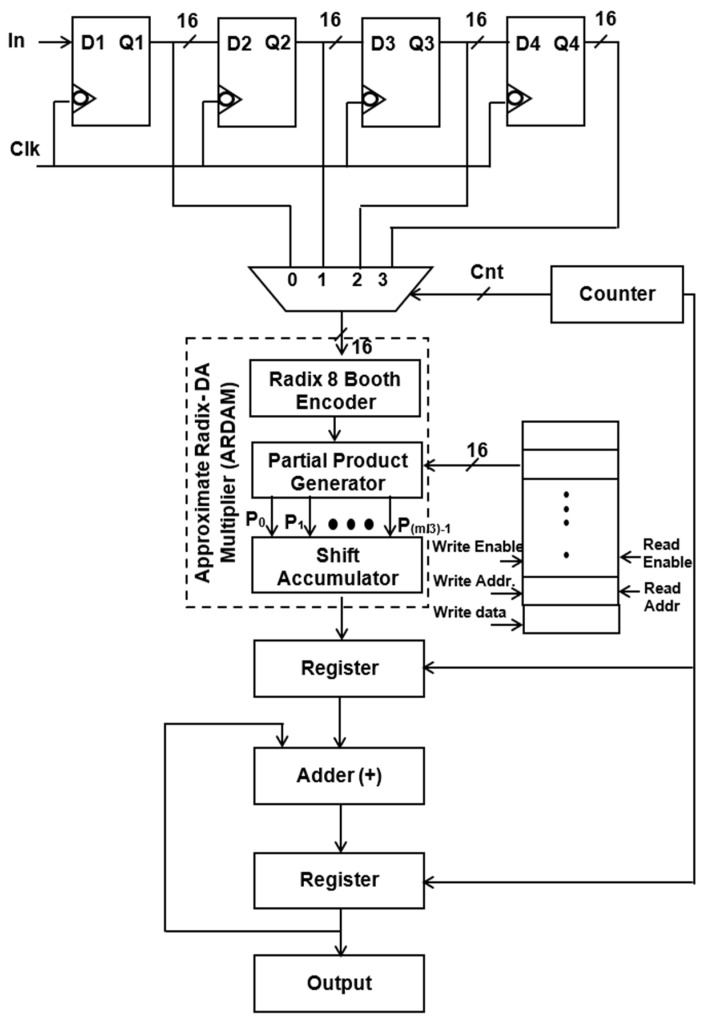
Structure of single ARDAM-based four-tap FIR filter (R-8).

It is evident from Equation (1) that the *N*-tap FIR filter needs *N* aggrandize and *N*−1 add units. Multipliers account for the majority of the filter’s complexity. The number of multipliers rises as the number of taps does. To get around these problems, a more effective architecture is suggested, in which the entire filter is operated by a single multiplier and adder, and the requisite delay registers are added to provide the required output sampling rate of the filter. Typically, *N* amount of clocking is essential for the *N*-tap filter to finish its filtering process. Assume that the sampling rate of the output filter for a four-tap design is increased to four MSPS from the entering sample rate of one mega sample per second (MSPS). Data are entered employing delay units into the multiply block to perform filtering operations for each clock cycle, and the output is accomplished in four clocks. Figure 1 and Figure 2 display the four-tap realization that incorporates the prevalent use of a sole MAC utilizing radix-4 (R-4) and radix-8 (R-8) schema. By lingering the input by one clock cycle, four delay registers are used to inject the eight-bit sample into a 4:1 MUX. Data selection across registers and multiplication operations are carried out using the 4:1 multiplexer. The counter increases the “cnt” enable, which selects the 4:1 MUX select lines. The counter chooses this number for the multiplication operation after delaying the input data by one clock interval for the first clock cycle. Similarly, using the counter’s “cnt” enable signal, further inputs are lingered in individual storage units and then moved into multiply blocks in every clock. To multiply data for each clock cycle, matching filter coefficients are injected and stored in RAM by sending the counter the commands “read enable” and “read address”.

Instead of storing the filter coefficients in registers, a 256 × 8-bit RAM is employed to hold them, which helps in minimizing transition activity. Here, the “read enable” parameter is used to retrieve the data from each RAM address. As soon as “write enable” is approved, and if the number of taps is enlarged, then the relevant details are kept in the RAM storage unit. For a 256-tap filter, the RAM can hold a maximum of 256 coefficients. Among these, the four-tap FIR filter uses just the top four sites when writing enable is reset to zero. A single generic counter selects the accumulator operation, multiplexer selects lines, and coefficient memory address arrays. The functioning of the proposed structure is also fortified by introducing the two approaches, Approximate DA-based radix-4 along radix-8, which are constructed in the multichannel filtering that significantly diminishes the longest path time as well as design complexity.

### 2.1. Approximate Distributed Arithmetic Design

As a p-bit fixed point realization, the longest route delay for the conventional filtering is (T_MUL_ + T_ADD_), where T_MUL_ and T_ADD_ are the longest route linger delays of a multiply and add block, correspondingly. As a result, this long latency limits the input signal’s sampling rate. The diminution of latency to obtain a higher output processing rate with adequate less space and power loss is a key component of the suggested FIR filter utilizing DA. According to the findings in [19], truncating the input operands reduces essential fixtures for add and multiply units more significantly than truncating the partial product. Therefore, to gain savings in the partial product creation, truncation is performed on the input operands. To preserve circuitry for PP aggregation, the majority of current designs are predicated on the cut-short information of the PPs [21]. Since these multipliers need every bit of the input operands, memory is not decreased for storage needs. On the other hand, in the deliberation with a huge information set, memory takes up a lot of space and power. Furthermore, reaching a high throughput requires effective data transmission [22]. Fixed-width multipliers [23] have made considerable use of truncation, which is an adept way to optimize power and area for the proximate computing units with confined preciseness [24]. To preserve circuitry for PP aggregation, the majority of alive formations are predicated on the curtailment of the PPs. Since these multipliers need every bit of the input operands, memory is not decreased for storage needs. Furthermore, reaching a high throughput requires efficient data transfers [25]. For a fixed-point implementation, relative computational units are, therefore, taken into consideration. For approximation arithmetic circuits, truncation is an adept way to reduce area and power with a minimal loss of accuracy [24], which is why it is widely utilized in fixed-width multiplier design [23,25]. In addition, to achieve savings in the PP’s creation, truncation is performed to the input operands. An additional popular filter design schema [26] utilizes the multiplexer-oriented manipulation sharing approach (MUX). The main concept is to extend the MUX-oriented procedure by further dispersing the error compensation and efficient processing over all taps to diminish circuit complication and power binge. In real-world applications, the filter coefficients are frequently modified serially. An identical module is recommended to decode the coefficients when reconfigurability is adopted to avail reduced complex realization.

Recently, advancements have been made in the field of adaptive FIR filter architectures, such as the peerless usage of DA-oriented filter framework for radix-4 and radix-8 manipulation [27], potent implementations of individual MAC-utilized filter realization for separate element analysis [28], and reconfigurable architectures for canceling unwanted components in auditory environments using sole MAC Adaline framework [29]. These advancements provide the foundation for further exploration into efficient multichannel FIR filter designs.

#### 2.1.1. Approximate Radix-4 Multiplier

The representation of a weightiness $w_{i}(n)$ in two’s complementing approach is

$$w_{i}\left(n\right)=-w_{i}^{m-1}\left(n\right)2^{m-1}+\sum_{j=0}^{m-2}w_{i}^{j}\left(n\right)2^{j}$$

where ${w_{i}}^{j}(n)$ depicts the *j*-th lower position detail of $w_{i}(n)$, and *m* indicates broadness in format. For the gratification of determined reasoning, $w_{i}(n)$ is specified as a whole number, and feasibly, it is quickly converted into a fixed-point format by adopting an appropriate shift. As indicated in Table 1, four bits of $w_{i}(n)$ are clustered using radix-4 Booth encoding.

Then, $w_{i}(n)$ is represented by Equation (2)

(2) $$\begin{array}{c} w_{i}\left(n\right)=\sum_{j=0}^{(m/2)-1}\left(-{2w}_{i}^{2j+1}\left(n\right)+w_{i}^{2j}\left(n\right){+w}_{i}^{2j-1}(n)\right)2^{2j}\\ ={\sum_{j=0}^{(m/2)-1}\overset{-}{w}}^{j}(n)2^{2j} \end{array}$$

where

$${w_{i}}^{-1}=0,{{\overset{-}{w}}_{i}}^{j}(n)=-2{w_{i}}^{2j+1}(n)+{w_{i}}^{2j}(n)+{w_{i}}^{2j-1}(n)$$

$${{\overset{-}{w}}_{i}}^{j}\left(n\right)\in \{-2,-1,0,1,2\}$$

**Figure 3:**
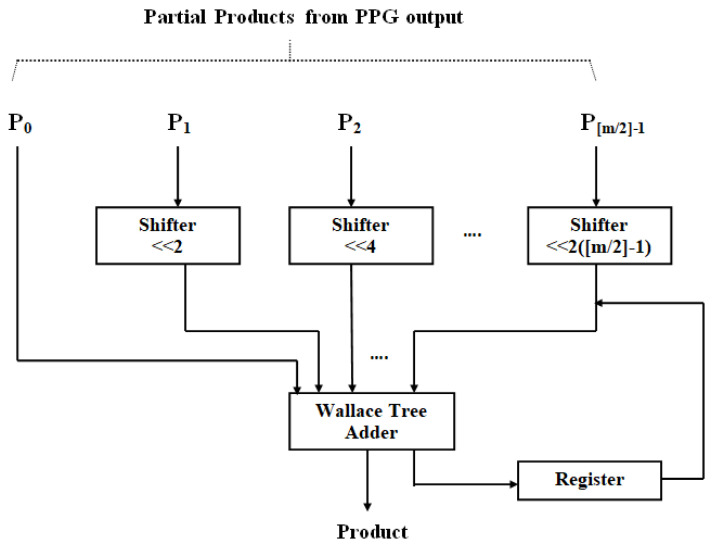
Radix-4 partial product generation.

In *pp*(*n*), there are fewer partial products than in a traditional DA architecture. Because of the radix-4 Booth method, [m−m/2]=m/2. Consequently, the number of aggregations needed to obtain y(n) is lowered to around half. The suggested multiplier module utilizing DA is displayed in Figure 3. Because a high-order filter has a considerable size cost, no LUT is employed in this design. Consequently, the PP terms (*PP_ij_*) are made and created through online means. The data w(n) and x(n) are first adjusted together with the truncate process. Moreover, to evaluate the result, radix-4 Booth recoding, PP creator, and nearby recoded add [24] are adopted to produce the PP terms (*PP_ij_*), where i=0, 1,…, M−1 and j=0, 1,…, [m/2]−1.

According to Table 1, the weight $w_{i}(n)$ is encoded every three bits (with a one-bit overlap) into a single value, ${{\overset{-}{w}}_{i}}^{j}(n)$ (that is 0, ±1, and ±2). This is accomplished by employing the Radix-4 Booth encoding scheme. Partial products PPij are produced by (10), using the PP’s creation unit and the approximation-based modified coded add block (to yield 2×(n−1)). The Wallace trees then aggregate the PP terms. The suggested design eliminates the DA-related final adder delay in the multiplier. Furthermore, the suggested technique is significantly faster because it makes use of Wallace trees. Ultimately, the near-close calculation in the PP’s creation and aggregation yields a momentous curtail in the design’s size and power consumption.

#### 2.1.2. Approximate Radix-8 Multiplier

A weightness $w_{i}(n)$ represented in two’s complementing approach is

$$w_{i}(n)=-{w_{i}}^{m-1}(n)2^{m-1}+\sum_{j=0}^{m-2}{w_{i}}^{j}(n)2^{j}$$

where ${w_{i}}^{j}(n)$ specifies the *j*th lower position component of $w_{i}(n)$, and m denotes the broadness of the format. For the gratification of determined reasoning, $w_{i}(n)$ is specified as a whole number, and feasibly, it is quickly modified into a fixed-point format by adopting a relevant shift. As indicated in Table 2, four bits of $w_{i}(n)$ are combined using radix-8 Booth encoding. Hence, $w_{i}(n)$ is given by the Equation (3)

(3) $$\begin{array}{c} w_{i}(n)=\sum_{j=0}^{(m/3)-1}(-2^{2}{w_{i}}^{3j+2}(n)+2{w_{i}}^{3j+1}(n)+{w_{i}}^{3j}(n)+{w_{i}}^{3j-1}(n))2^{3j}\\ ={\sum_{j=0}^{(m/3)-1}\overset{-}{w}}^{j}(n)2^{3j} \end{array}$$

where

$${w_{i}}^{-1}=0,{{\overset{-}{w}}_{i}}^{j}(n)=-2^{2}{w_{i}}^{3j+2}(n)+2{w_{i}}^{3j+1}(n)+{w_{i}}^{3j}(n)+{w_{i}}^{3j-1}(n)$$

$${{\overset{-}{w}}_{i}}^{j}\left(n\right)\in \{-4,-3,-2,-1,\;0,\;1,\;2,\;3,\;4\}$$

Polarity expansion is utilized when the broadness of the recoded input is lower than 3xm/3. The response of *y*(*k*) is indicated, as given in Equation (4)

(4) y(k)=x(k).w(k)=δx(k)w(k)

where

$$\bar{w}(n)=\left[\begin{array}{ccc} {{\bar{w}}_{0}}^{0}(n) & {{\bar{w}}_{1}}^{0}(n)\ldots & {{\bar{w}}_{M-1}}^{0}(n)\\ {{\bar{w}}_{0}}^{1}(n) & {{\bar{w}}_{1}}^{1}(n) & {{\bar{w}}_{M-1}}^{1}(n)\\ {{\bar{w}}_{0}}^{\left\lceil \frac{M}{3}\right\rceil -1}(n) & {{\bar{w}}_{1}}^{\left\lceil \frac{M}{3}\right\rceil -1}(n) & {{\bar{w}}_{M-1}}^{\left\lceil \frac{M}{3}\right\rceil -1}(n) \end{array}\right]$$

$$pp_{j}(n)=\sum_{i=0}^{M-1}{w_{i}}^{j}(n)x(n-i)=\sum_{i=0}^{M-1}PP_{ij}$$

$$\delta =[2^{0},2^{3},\ldots ,2^{3(m/3)-3}]$$

$x(n)={\left[x\left(n\right),x\left(n-1\right),\ldots ,x\left(n-M+1\right)\right]}^{T}$, $pp(n)=\bar{w}(n)x(n)$ and $y\left(n\right)=\delta \cdot pp(n)$. where $pp\left(n\right)={\left[pp_{0}\left(n\right),pp_{1}\left(n\right),\ldots ,pp_{m/3-1}(n)\right]}^{T}$ Examining a traditional DA structure, the amount of PPs in *pp*(*n*) is diminished by approximately [*m* − *n*/3] = 2m/3 because of the incorporation of the radix-8 Booth formulary. Therefore, the needed agglomeration to achieve *y*(*n*) is downsized by around 2/3.

**Figure 4:**
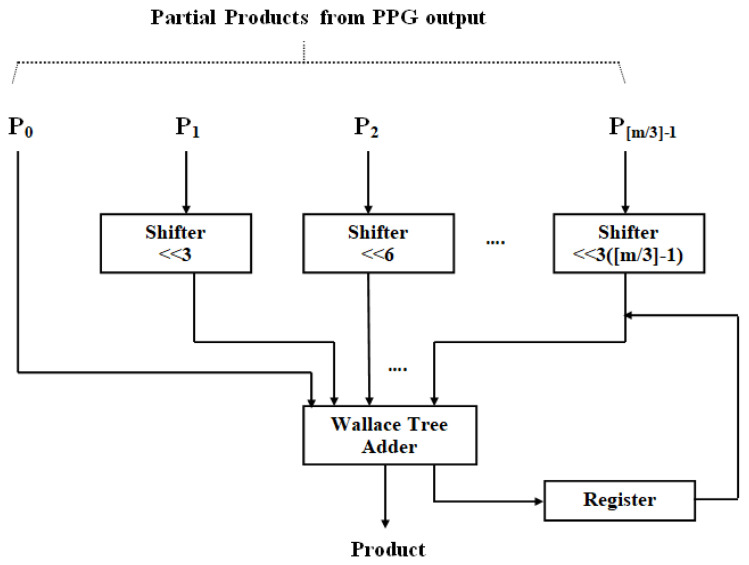
Radix-8 partial product generation.

The suggested multiplier unit utilizing DA is displayed in Figure 4. Because a high-order filter has a considerable size cost, no LUT is employed in this design. Consequently, the PPs (*PP_ij_*) are constructed and generated online. The data *x*(*n*) as well as *w*(*n*) are first adjusted together with the truncate process. Moreover, to evaluate the result, radix-8 Booth recoding, PP creator, and nearby recoded add [29] are adopted to produce the PP terms (*PP_ij_*), where i=0, 1,…,M−1 and j=0, 1,…,[m/3]−1.

Radix-8 Booth recoding process involves encoding a group of four bits that exist in the weightness $w_{i}(n)$ (one position imbricate) to a term ${{\overset{-}{w}}_{i}}^{j}(n)$ (that is 0, ±1, ±2, ±3, and ±4), as listed in Table 2.). The PP’s creator and the near close modified coder-based add unit (to produce 3 × (*n* − 1)) are utilized to create *PP_ij_*, as per (10). Further, the PPs are aggregated with the Wallace tree approach.

The suggested structure eliminates the need for eventual add unit delay in the multiply block because of the existence of DA. Furthermore, the suggested technique is significantly faster because it makes use of Wallace trees. Likewise, the near-close evaluation of the PP’s creation and aggregation provides a momentous curtail in the design’s size and power consumption.

## 3. Propounded Multichannel Approximate DA Utilized Filter Design (M-ADAFD)

TDSP is extensively used in intercommunication domains like SDR, as well as acoustics, to simultaneously receive signals from several stations; multichannel manipulation is, therefore, crucial for the dependability and competent process of SDR technology. The Time Division Multiplex approach, which uses a single set of hardware resources to optimize the logic resources across many data streams, is a considerably more hardware-efficient approach for optimizing logic resources. For the most effective use of device utilization, the M-ADAFD structure uses a time division mechanism to divide the logic resources among several sample streams. Xilinx Inc. (San Jose, CA, USA) discusses the traditional TDM-based multichannel FIR filter, wherein filtering is achieved for several incoming channels by applying a different filter structure for individual channels. P multipliers and P-1 adders are needed for a P-tap filter having multiple channels. If a channel has a sampling frequency of fs, then executing a bunch of M samples requires a sampling frequency of fs/M for an M-channel filter.

**Figure 5:**
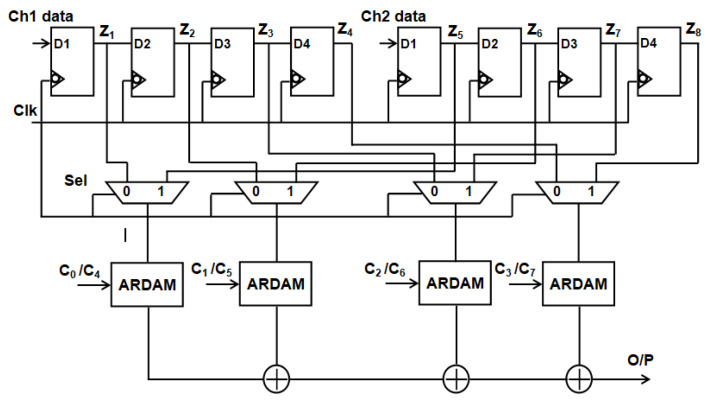
Traditional two-channel structure of ADA MAC FIR filter.

Examining the typical two-channel, four-tap filter structure depicted in Figure 5, data for channels 1 and 2 are obtained concurrently. The eight-bit input sample that is kept in channel 1 and channel 2 registers D1 through D4. Data from channels 1 and 2 are each pumped into the 2:1 MUX. Here, the select line for two-channel operation is the “clk” signal. Coefficients that are kept in the registers are multiplied by the multiplexed single-channel data. To obtain the filter output, the products are added. Three adders and four multipliers are needed to compute this filter. The complexity and power consumption increase with the number of channels and taps used.

To address the challenges associated with the traditional framework, the M-ADAFD is suggested. The suggested M-ADAFD is built utilizing one multiply and add device independent of taps and channels used. Delay registers are inserted to figure out the filter operable frequency. M * N clocking is enforced to compute the outcome of the filter having M channels and N taps. Also, this is achieved with N clocks.

**Figure 6:**
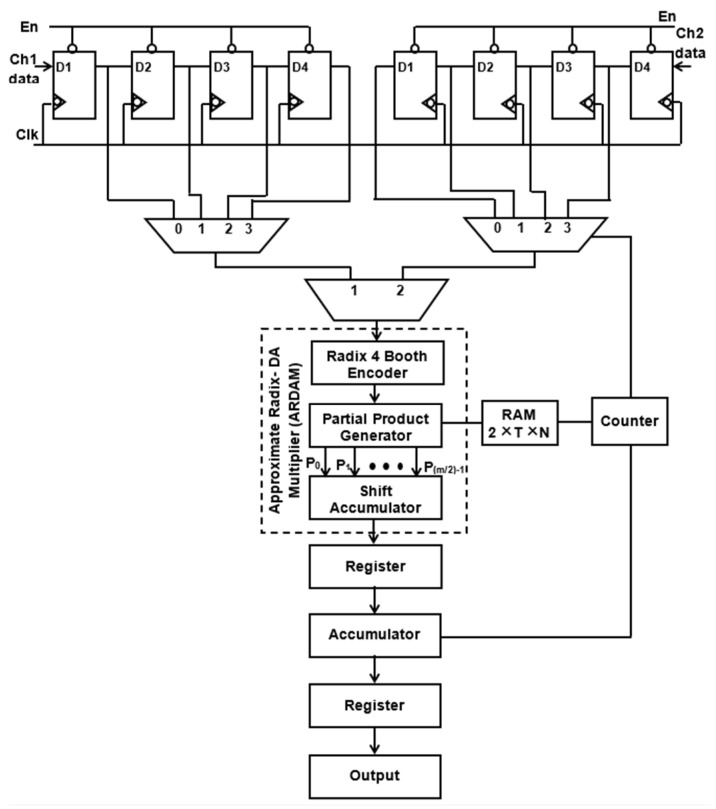
Suggested structure of two-channel ADA MAC FIR filter (R-4).

**Figure 7:**
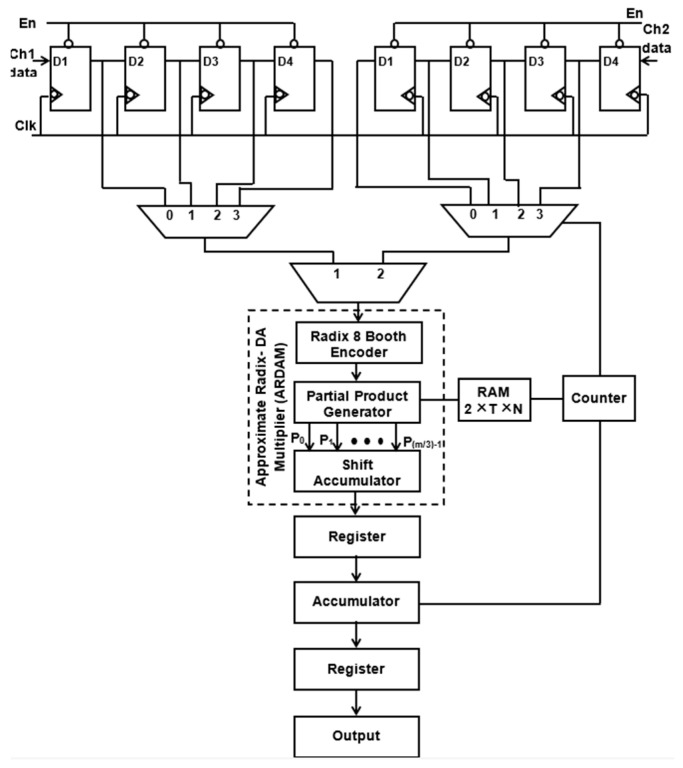
Suggested structure of two-channel ADA MAC FIR filter (R-8).

Figure 6 and Figure 7 depict the suggested M-ADAFD for a two-channel four-tap FIR filter with R-4 and R-8. In this framework, four clocks are appropriate to process an individual channel, and therefore, a total of eight clocks are enforced to realize the complete design. The first batch data for channels 1 and 2 are initially fetched concurrently into the D1 delay for the individual channel. They are further picked using two distinct 4:1 MUXes and delayed by one clock cycle. The enable signal from a counter is used to pass each of these multiplexers’ outputs to a multiplier individually. To perform a filter operation, channel 1 data are injected into the multiplier for the first four clock cycles, and the response is attained within this duration itself. The data are chosen for two channels, each with four lines, using an 8:1 multiplexer. In the register, the multiplied and aggregated multiplexed single-channel data are reserved. Following this process, the subsequent bunch of data stream from channel 2 is retrieved and enumerated. After that, MAC is reset to make room for the subsequent round of channel information. The coefficient is saved in a generic 256 × 8-bit RAM, of which only 8 × 8 bits are utilized, depending on the wielded taps and channels. After setting write enable, RAM holds the coefficients as two bunches of four positions, and when resetting the write enable, the further RAM positions are set to 0. The accumulator operation, coefficient memory address arrays, and multiplexor select lines are all accessed using a single generic counter. Similarly, an eight-tap two-channel design necessitates 16 clocks. To avail the imperative operating frequency, N-taps, including M channels, each built with sole multiply and add module by interjecting N delay elements and the RAM, hold the vital coefficients.

## 4. Results and Dissertation

This section confers the responses attained for the investigated single-channel Approximate DA Utilized Filter Design (S-ADAFD) and M-ADAFD compared with the already reported research works. Parameterizable Verilog cores are utilized to build the recommended architecture for the following specifications. Using the Hamming Window approach, a lower frequency operating filter network is designed by considering 450 kHz cutoff frequency (Fc) together with 1000 kHz sample frequency (Fs). Both S-ADAFD and M-ADAFD are developed using the Xilinx Virtex-5 device and the Cadence tool, adopting a 0.18 µm technological node.

### 4.1. Proposed Single Channel Approximate DA Utilized Filter Design

**Figure 8:**
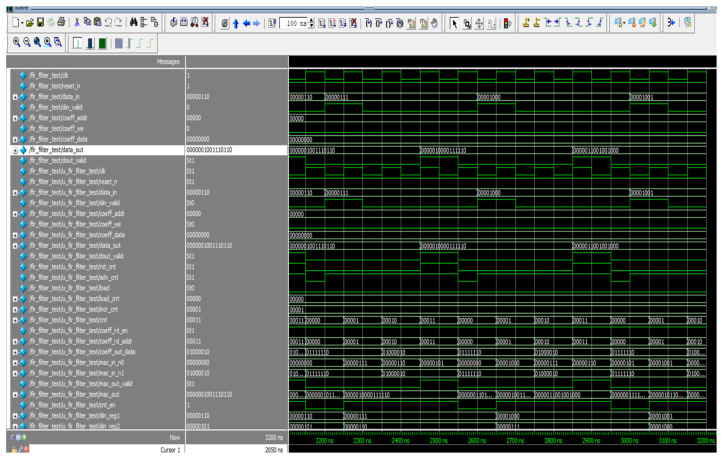
Simulated result of S-ADAFD.

Figure 8 depicts the simulated response of the proposed S-ADAFD. The key information for S-ADAFD is din_valid and data_in, and the corresponding results are obtained as data_out together with data_out_valid. In RAM, the coefficients are saved like Coeff_data. Time-shared multiply realization throughout a sole MAC unit is employed to establish the filtering process that mandates four clocks to construct the four-tap FIR filter.

**Figure 9:**
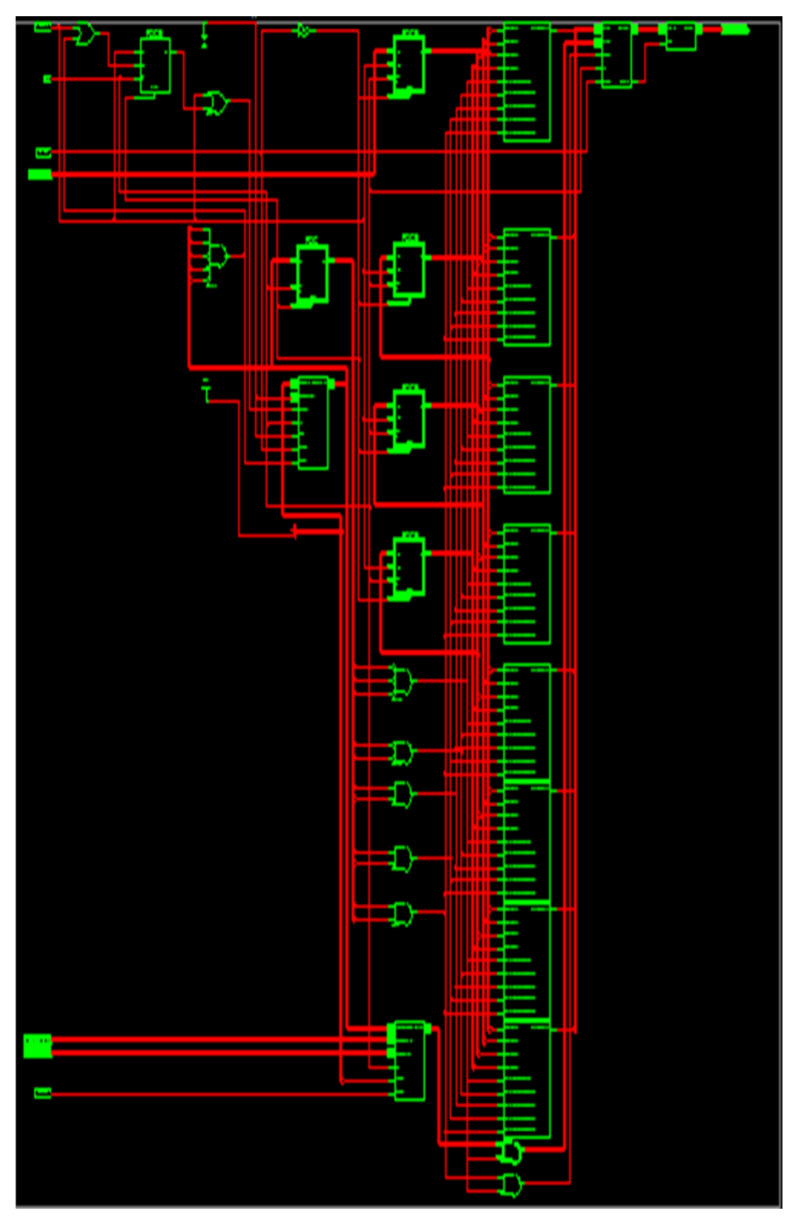
Netlist view of ADA_MAC filter.

As a result, the entering signal sample frequency is four times smaller than the working frequency of the output filter. When din_valid is provoked, the data are given every four clocks to correlate the ensuing sample frequency. When data_out_valid is facilitated, the data_out is attained after the duration of 20 clocks (200 ns) (150 ns for manipulating the eight-bit data inputs together with 50 ns latency). The RTL illustration of S-ADAFD is displayed in Figure 9. RAM usage, MAC, and a single-channel FIR filter multiplexer are crucial parts of calculating the area.

The Xilinx Virtex-5 XC5VSX95T-1FF1136 FPGA device is adopted to fetch the results of the suggested FIR filter design. It is constructed utilizing two multiplier schemes: (i) Approximate DA-Radix-4 and (ii) Approximate DA-Radix-8 Multipliers. Table 3 presents an analysis of the FIR filter designs’ performance outcomes. The single MAC core design used to achieve the suggested single-channel FIR filter incorporates a time-sharing technique that works regardless of the number of taps. The size increases slightly as the number of filter taps increases because multiplexer logic and register complexity increase. Two techniques—Approximate DA-Radix-4 and Approximate DA-Radix-8—help further reduce the multiplier’s complexity.

**Figure 10:**
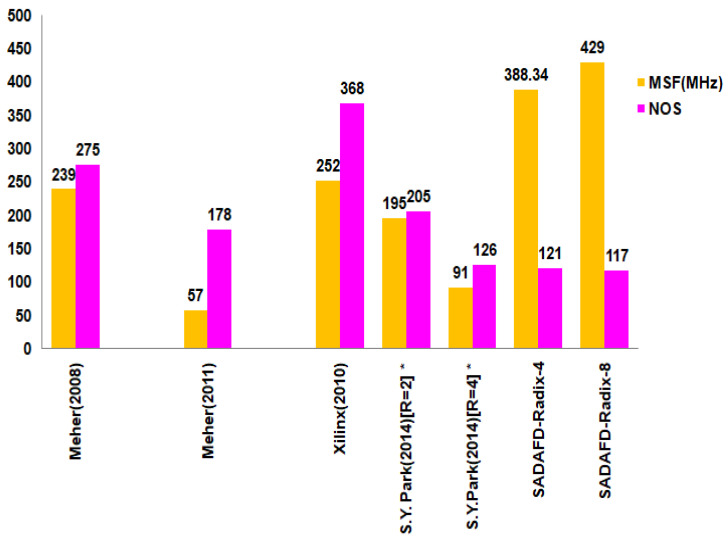
Characteristics of distinct realizations.

The synthesized responses of sixteen-tap filter topologies are compared together with current structures in Table 4. The Xilinx Virtex-5 FPGA device is used in the synthesis of both the suggested and current architectures. Table 4 lists the following parameters: number of slice registers (NSREG), minimum sample period (MSP), number of slices LUTs (NSLUT), number of slices (NOS), and maximum sample frequency (MSF). When examining the reported structures, the speed performance of the propounded Acc radix-4 and single-channel FIR filter with radix-8 architecture exhibits improvements of 35% and 41%, respectively. Comparing the slice-delay product to current architectures, the improvements are 70% and 74%, respectively. By inserting pipelined registers between the multiplier and adder, the suggested architecture maximizes sampling frequency while simultaneously maximizing space efficiency through the utilization of a sole MAC, including shared resources. The performance characteristics of the distinct structures are seen in Figure 10.

The synthesized summary of eight-tap filter topologies is examined with the current realizations and is quoted in Table 5. Using a Xilinx Virtex IV FPGA to target the Xc4vf100 device, this work was synthesized and implemented to acquire the highest operation frequency and actual utilization of hardware resources. Because the data retrieval duration from the LUT is quicker over the typical multiply process, the devised radix-4 and radix-8-based S-ADAFD offers a 28% speed enhancement over the already presented framework.

The hardware and time complexity of the S-ADAFD is listed in Table 6. The filter structure has a delay that takes five clock cycles. Four clock cycles are needed for MAC operation: two are needed for registering the ensuing sample, one is needed for multiply, and then another for aggregation. Lastly, the output must be registered after one clock cycle. For this architecture, *N* + 5 total registers are involved for the *N*-tap structure. Regardless of the filter taps, sole multiply and add elements are needed for filter operation due to the resource-sharing concept.

The proposed MAC designs and the existing reconfigurable architecture [26] are compared in Table 7. In comparison with the reported architectures, it offers significant performance and space savings through the use of Approximate DA-based MAC techniques. The reason for the operation’s increased speed is that just the product that is stored in the LUT is obtained.

### 4.2. Proposed Multichannel Approximate DA Utilized Filter Design

**Figure 11:**
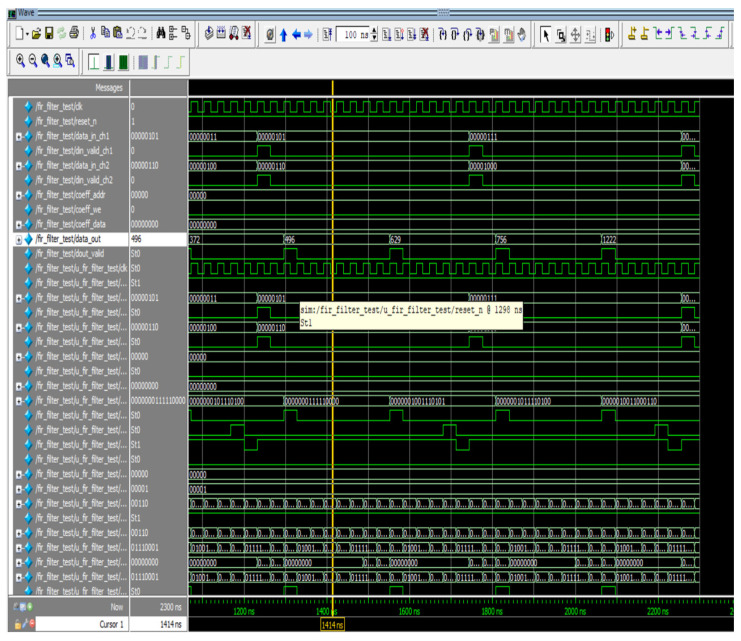
Simulation waveform of M-ADAFD.

The outcomes of individual channels are attained sequentially for a multichannel FIR filter. In this study, we examined a two-channel, eight-tap FIR filter for waveform analysis. This design is implemented using the same low-pass filter parameters covered in Section 4.1. Figure 11 displays the simulation waveforms for the proposed multichannel FIR filter. In this architecture, data (x) refers to channel 1, and data (x1) indicates channel 2, which are retrieved subsequently, and the responses (filter_out) are availed after a certain duration as a result of parallel MAC structures. Data_in_ch1, along with data_in_ch2, are fetched concurrently in Figure 11, and the outputs are obtained at data_out in a sequential order. Because of the individual MAC, the data_out of channel 1 is produced with a latency of eight clocks. Channel 1 and Channel 2 have sampling frequencies of fs and fs/2, respectively. Here, the sampling frequency is sixteen times greater than the ensuing sample frequency, and sixteen clocks are needed to establish the response of both channels. As a result, data are fetched once every 16 clock cycles for channels 1 and 2. After 2080 ns, the filter response (data_out) is acquired (580 ns latency for both channels and 1500 ns for carrying out eight-bit input).

**Figure 12:**
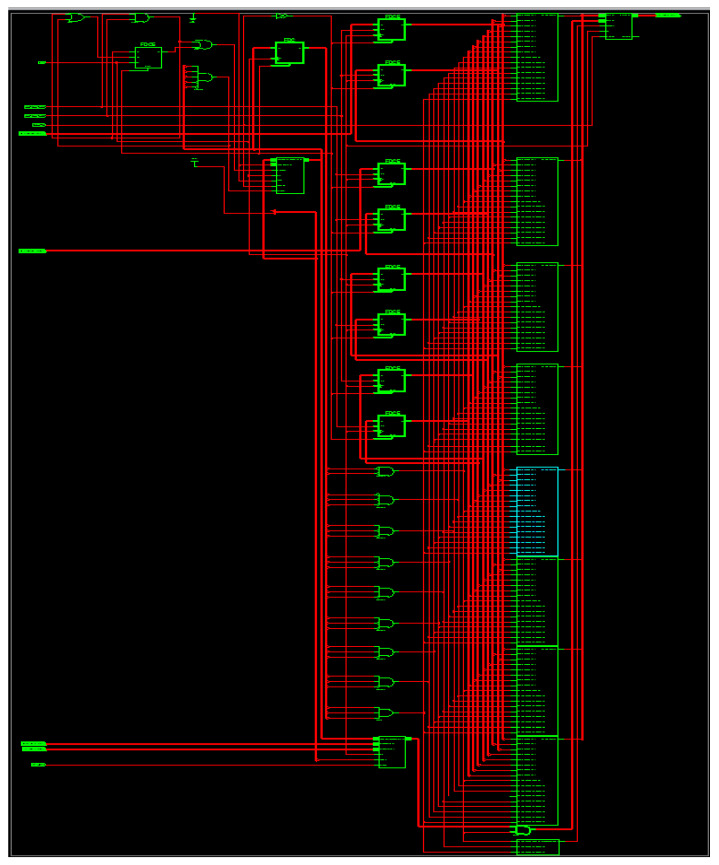
RTL schematic of M-ADAFD.

The RTL representation for the suggested M-ADAFD is displayed in Figure 12. Table 7 provides an analysis of the performance of the proposed M-ADAFD that is produced using a Xilinx Virtex-5 FPGA chip. The TDM concept optimizes the use of logical elements. From Table 8, it is evident that when the number of taps grows, the area of both structures increases somewhat. The rising complexity of delay registers and multiplexer logic is the cause of the expanding area. When compared to the Approximate DA-radix-4-based design, the DA-radix-4-based multichannel architecture achieves optimal performance due to the reduction of partial products.

The results of the investigated 16-tap M-ADAFD are correlated with those of the traditional multichannel realization utilizing the 0.18 µm CMOS technology Cadence RC compiler and Xilinx VIRTEX-5 FPGA, as shown in Table 9 and Table 10. It is evident from Table 8 and Table 9 that the development of a multichannel FIR filter based on TDM is very effective at using logic resources. In comparison to traditional multichannel realization, it is deduced that the suggested M-ADAFD occupies less NOS when implemented in FPGA. Table 10 shows that, in comparison to the traditional multichannel realization in ASIC, a significant area decrease is obtained for the suggested multichannel filter architecture. The synthesized output of six-channel eight-tap filter topologies is compared over the current framework utilizing the Xilinx Virtex-5 FPGA device and is specified in Table 9. When compared to the current structure, the suggested structures’ slice-delay product demonstrates a 25% improvement. The single MAC core architecture of the suggested multichannel FIR filter topologies reduces complexity, while the parallel MAC architecture of the current architecture lowers speed performance.

The major benefaction of the devised research is realizing an effective single and multichannel FIR filter framework utilizing individual MAC units, proximate DA, and pipelining techniques. Time-shared separate MAC incorporation and utilizing proximate DA diminishes the complex design to a considerable level when compared with the parallel MAC-based technique in other works of the literature. Furthermore, the pipelining offers speed embellishment. The parameter area (resources) and complexity are significantly lower than the prevailing works. Moreover, the speed of the propounded framework was identified to be remarkably enhanced while correlating with the existing work.

TDM in a multichannel Finite Impulse Response (FIR) filter offers several advantages, especially when it comes to optimizing hardware resources and improving performance. Time Division Multiplexing in multichannel FIR filters offers advantages in terms of hardware efficiency, scalability, power savings, cost reduction, and simplified system design. It is particularly useful in applications where hardware resources are limited, but multiple signal channels need to be processed concurrently.

The TDM offers benefits such as resource sharing and cost minimization, and its impediments include increased latency, stringent timing constraints, potential performance bottlenecks, and more complex control logic. These limitations must be carefully envisaged, especially in applications that mandate minimal latency, larger bandwidth, or asynchronous data processing. The adaptability of a multichannel FIR filter to different input signals is largely driven by the inherent flexibility of FPGAs and the design choices made for the filter structure. FPGAs allow the customizable coefficients to be readily reprogrammed, enabling the filter to be tailored to different signal characteristics.

Advanced FPGAs support partial reconfiguration, meaning that a portion of the filter formulary can be dynamically modified during operation. This facilitates the filter to switch between distinct modes of operation. The adaptability extends to distinct bandwidths. The FIR filter design can be adjusted to process narrowband, wideband, or ultra-wideband signals. This is particularly useful in applications like SDR, where signal types can change dynamically. FPGA platforms are widely used in SDRs due to their high processing power, parallelism, and reconfigurability. Implementing multichannel FIR filters on FPGAs can meet the demands of real-time signal processing in SDR systems.

## 5. Conclusions

This research work presents the filter architecture to support multichannel communication together with a single channel. Two distinct realizations are incorporated in the proposed work, namely S-ADAFD and M-ADAFD. The proposed realizations are handled with the sole MAC functioning unit along with a time-sharing scheme. It is demonstrated that the resource-sharing multiplier and adder can significantly lower hardware costs. The suggested single-channel FIR filter architectures (with DA-Radix-4 and DA-radix-8) perform better because of speed improvement and area curtailment when compared to the results of the existing architectures. When examining already reported designs, the speed performance of the suggested structures demonstrates improvements of 35% and 41%, respectively. Comparing the suggested DA-Radix-4 structure to existing architectures, the slice-delay product exhibits 70% improvement, and the DA-Radix-8 structure offers 74% improvement.

A single-channel FIR filter with a maximum input sampling frequency of 429 MHz can be implemented on an FPGA, thanks to the suggested structure. Higher speed and low complexity are attained by DA-Radix-4 and Da-Radix-8 architectures in multichannel FIR filters, and when compared to the current structure, the suggested structures’ slice-delay product improves by 30%. For real-time signal processing applications, the suggested multichannel architectures are, therefore, a good substitute for the current architectures because of their low complexity and effective reprogrammability.

## Figures and Tables

**Table 1:** Radix-4 Booth encoding algorithm.

$w_{i}{\left(n\right)}^{2j+1}$	$w_{i}{\left(n\right)}^{2j}$	$w_{i}{\left(n\right)}^{2j}-1$	${{\overset{-}{w}}_{i}}^{j}(n)$
0	0	0	0
0	0	1	+1
0	1	0	+1
0	1	1	+2
1	0	0	−2
1	0	1	−1
1	1	0	−1
1	1	1	0

**Table 2:** Radix-8 Booth encoding algorithm.

$w_{i}{\left(n\right)}^{3j+2}$	$w_{i}{\left(n\right)}^{3j+1}$	$w_{i}{\left(n\right)}^{3j}$	$w_{i}{\left(n\right)}^{3j-1}$	${{\overset{-}{w}}_{i}}^{j}(n)$
0	0	0	0	0
0	0	0	1	+1
0	0	1	0	+1
0	0	1	1	+2
0	1	0	0	+2
0	1	0	1	+3
0	1	1	0	+3
0	1	1	1	+4
1	0	0	0	−4
1	0	0	1	−3
1	0	1	0	−3
1	0	1	1	−2
1	1	0	0	−2
1	1	0	1	−1
1	1	1	0	−1
1	1	1	1	0

**Table 3:** S-ADAFD performance findings.

Performance Measures	ADA-Radix-4			ADA-Radix-8		
Device Used	XILINX VIRTEX-5 FPGA					
Filter Length	4	8	16	4	8	16
Number of Slices Registers (Available 58880)	122 (0.20%)	149 (0.25%)	216 (0.36%)	114 (0.21%)	143 (0.27%)	208 (0.38%)
Number of Slices LUTs (Available 58880)	436 (2.17%)	452 (21.77%)	472 (0.22%)	432 (0.74%)	442 (0.75%)	468 (0.81%)
Number of Fully- used Slices (NFUS)/Number of Used Slices (NUS)	94 (8.5%)	108 (7.3%)	121 (7.82%)	93 (20.8%)	105 (21.1%)	117 (20.7%)

**Table 4:** Comparison of alternative architectures and the suggested S-ADAFD.

Realization	MSP (ns)	MSF (MHz)	NOS	NSREG	NSLUT	Slice-Delay Product
Meher [11]	4.17	239	275	688	833	1147
Meher [17]	17.35	57	178	412	267	4632
Xilinx [19]	3.96	252	368	970	806	1457
Sang Yoon Park [21] [R = 2] *	5.11	195	205	671	517	1048
Sang Yoon Park [21] [R = 4] *	10.91	91	126	397	277	1375
Propounded S-ADAFD-Radix-4	2.575	388.34	121	216	472	311.575
Proposed S-ADAFD-Radix-8	2.326	429	117	208	468	272.12

* R indicates the number of TDM slots.

**Table 5:** Comparison of suggested S-ADAFD with already reported works.

Realization	MSP (ns)	MSF (MHz)	NOS	NSREG	NSLUT
Raghidi [22]	2.713	302.253	287	196	379
Investigated S-ADAFD-Radix-4	2.67	374.53	285	180	349
Proposed S-ADAFD-Radix-8	2.55	392	297	184	361

**Table 6:** Hardware and time complexities of ADA-FIR filter.

Proposed S-ADAFD	Complexity		
Taps	4	8	16
Multiply Element Required	1	1	1
Registers Required	9	13	21
Adders Required	1	1	1

**Table 7:** Implementation results of filter structures. (Device: Xilinx ZYNQ-XC7Z020-1CLG84C FPGA device).

Parameters	Zhang L, Rao C, Lou X [26]	Proposed MAC-Radix-8	Proposed MAC- Radix-4
Number of Slices	1014	212	224
Delay (ns)	1.81	1.61	1.73
Slice LUT	936	848	856

**Table 8:** Findings for 16-tap M-ADAFD.

Specification	Traditional Structure				Propounded M-ADAFD			
	Acc-Radix-8		Acc-Radix-4		Acc-Radix-8		Acc-Radix-4	
Used Channel	2	4	2	4	2	4	2	4
Slices Registers Available 58880	491	745	386	641	356	574	578	581
Slice LUTs Available 58880	2100	2161	3501	3532	515	658	575	676
NFUS /NUS	340 (15.5%)	516 (21.8%)	224 (5.8%)	315 (7.3%)	161 (21.1%)	174 (21.1%)	173 (10.46%)	187 (12.15%)
Number of Slices Used Available 58880	2250	2317	3700	3870	727	520	578	581
MSP (ns)	0.63	1.2	0.857	1.4	2.523	2.84	2.65	2.91
MSF (MHz)	1587	833,33	1166	714.28	396.35	352.11	377.35	343.64

**Table 9:** Devised M-ADAFD with existing work.

Design	MSP (ns)	MSF (MHz)	NOS	Slice-Delay Product
Xilinx Inc., (2005)	2.202	454	216	476
M-ADAFD-radix-4	2.10	476	157	329.70
M-ADAFD-radix-8	2.05	487.80	169	346.45

**Table 10:** Findings for 16-tap M-ADAFD using Cadence tool.

Parameter	Existing Design				Proposed Design			
	Acc-Radix-4		Acc-Radix-8		Acc-Radix-4		Acc-Radix-8	
No. of Channels	2	4	2	4	2	4	2	4
Area (mm^2^)	0.101	0.113	0.141	0.16	0.063	0.083	0.093	0.098

## Data Availability

The manuscript has no associated data.
